# First Trimester Prediction of Adverse Pregnancy Outcomes—Identifying Pregnancies at Risk from as Early as 11–13 Weeks

**DOI:** 10.3390/medicina58030332

**Published:** 2022-02-22

**Authors:** Alexandra Bouariu, Anca Maria Panaitescu, Kypros H. Nicolaides

**Affiliations:** 1 Department of Obstetrics and Gynecology, Filantropia Clinical Hospital, 011171 Bucharest, Romania; alexandra.bouariu@yahoo.com; 2 Division of Physiology and Neuroscience, Carol Davila University of Medicine and Pharmacy, 050474 Bucharest, Romania; 3 Harris Birthright Research Centre of Fetal Medicine, King’s College Hospital, London SE5 8BB, UK; kypros@fetalmedicine.com

**Keywords:** 11–13 weeks, first trimester, pregnancy outcomes, chromosomal abnormalities, structural defects, preeclampsia, gestational diabetes, preterm birth, placenta accreta spectrum, small for gestational age, fetal growth restriction

## Abstract

There is consistent evidence that many of the pregnancy complications that occur late in the second and third trimester can be predicted from an integrated 11–13 weeks visit, where a maternal and fetal assessment are comprehensively performed. The traditional aims of the 11–13 weeks visit have been: establishing fetal viability, chorionicity and dating of the pregnancy, and performing the combined screening test for common chromosomal abnormalities. Recent studies have shown that the first trimester provides important information that may help to predict pregnancy complications, such as preeclampsia and fetal growth restriction, stillbirth, preterm birth, gestational diabetes mellitus and placenta accreta spectrum disorder. The aim of this manuscript is to review the methods available to identify pregnancies at risk for adverse outcomes after screening at 11–13 weeks. Effective screening in the first trimester improves pregnancy outcomes by allowing specific interventions such as administering aspirin and directing patients to specialist clinics for regular monitoring.

## 1. Introduction

The early assessment of patient specific risks of pregnancy complications has the purpose to improve pregnancy outcome and to individualize the patient- and disease-specific approach. From 11–13 weeks onwards, pregnancies may be classified as being low-risk for pregnancy complications, with a substantially reduced number of medical visits, and high-risk, with close surveillance in specialists’ clinics. Low-risk pregnancy includes not only a low chance for a chromosomal abnormalities ratio after the first trimester combined screening test, that includes maternal history, fetal ultrasound assessment and serum biochemistry assessment of beta-hCG (beta human chorionic gonadotropin) and PAPP-A (pregnancy-associated plasma protein-A), but also a low chance of maternal pregnancy complications considered after assessing the chance for developing preeclampsia, fetal growth restriction and preterm delivery, by using maternal history characteristics, ultrasound aspects and the serum biochemistry of PAPP-A, soluble fms-like tyrosine kinase 1 (sFlt-1), and placental growth factor (PlGF) [1].

The aim of this manuscript is to review the methods available to identify pregnancies at risk for adverse outcomes after screening at 11–13 weeks. The pioneering work in this field was undertaken at King’s College Hospital, London, UK and it offers consistent scientific support to be adopted and applied whenever possible.

## 2. Detection of Chromosomal Abnormalities and Fetal Structural Defects

The traditional aims of the 11–13 weeks visit have been: establishing fetal viability, chorionicity and dating of the pregnancy, and performing the combined screening test for common chromosomal abnormalities. Gradually, as the technology improved, and with the increasing desire of those performing the ultrasound scans, a detailed anatomy check of the fetus was also introduced. The first trimester detection of many fetal anomalies is possible by applying a standardized examination protocol [2].

Chromosomal abnormalities or aneuploidies are a major cause of childhood disability and perinatal death. Therefore, the detection of aneuploidies represents an important part of the first trimester assessment. In cases of a high chance result for chromosomal abnormalities there is the possibility of invasive prenatal diagnosis and confirmation of the disorder [1]. The first trimester screening test can identify about 90% of fetuses with trisomy 21 (Down syndrome) and other major aneuploidies for a false-positive rate of 5%. The screening began by assessing the risk for aneuploidies using only the maternal age in the 1970s, then maternal serum biochemistry and fetal ultrasound examination was added in the 1980s, followed by using a combination of maternal age, fetal nuchal translucency (NT) thickness, maternal serum free beta-hCG and PAPP-A in the 1990s [3]. At the moment, additional markers such as the presence of nasal bone [4], the flow in the ductus venosus [5] and across the tricuspid valve [6] improve the detection rate of trisomy 21 to 96% [1].

Fetal anomalies were divided into first, those that should be always detectable in the first trimester, such as acrania, alobar holoprosencephaly, exomphalos and gastroschisis; second, those that are sometimes detectable, such as spina bifida and facial cleft, depending on the use of a standardized protocol for examination of the fetus; and third, those that are never detectable, such as the absence of the corpus callosum and congenital pulmonary airway malformation [2]. Using standardized views of the fetal anatomy in the first trimester, fetal abnormalities may be demonstrated at a routine fetal assessment (Table 1).

## 3. Screening for Preeclampsia

Extensive research has now established the feasibility of the first-trimester prediction of about 90% of cases of early preeclampsia (PE) before 32 weeks, 75% of preterm PE before 37 weeks, and 45% of term PE by combining maternal characteristics and medical history with maternal serum placental growth factor (PlGF), the uterine artery pulsatility index (UtA-PI) and mean arterial pressure (MAP) [7,8].

The maternal characteristics considered high risk factors for developing PE are a history of hypertensive disease in previous pregnancy, chronic hypertension, chronic kidney disease, autoimmune disease, diabetes mellitus, increasing maternal age and weight, long inter-pregnancy interval, a family history of PE, and black race.

A large multicenter study, the ASPRE trial, used the combined method of first trimester screening, utilizing maternal risk factors, the PlGF, UtA-PI and MAP, to identify a high-risk group for PE [7,8]. This high-risk group was then randomized to aspirin (150 mg/day) or a placebo from 12 to 36 weeks’ gestation. The trial showed that aspirin can prevent about 90% of early PE and 60% of preterm PE [9].

## 4. Screening for Small for Gestational Age (SGA)

A model that combines maternal characteristics and medical history with biochemical markers in the first trimester can predict about 60% of small for gestational age (SGA) neonates below the 10th centile born before 32 weeks’ gestation, and 80% of extremely low birth weight neonates resulting from pregnancies with preterm preeclampsia [10,11]. PlGF and PAPP-A, which are considered markers of impaired placentation, are predictive of SGA, serum PlGF being a better predictor of SGA with PE and having equal prediction as PAPP-A in predicting SGA without PE. The reason for the differences regarding PE and SGA prediction is that PAPP-A is mainly a regulator of insulin like growth factors, whereas PlGF is directly involved in angiogenesis, hence, closer in the pathophysiology of the altered angiogenesis of PE and SGA [12].

Early prediction allows early intervention and as shown in the ASPRE trial, the use of aspirin reduced the overall incidence of SGA <10th percentile by about 75% in babies born at <32 weeks’ gestation [13].

## 5. Screening for Preterm Birth

Preterm birth complicates around 10% of all pregnancies and is the main cause of neonatal death and neurological handicap in children.

The risk for spontaneous early preterm delivery is influenced by various maternal characteristics: it increases with maternal age, decreases with height, it is higher in women of African and South Asian racial origin, in cigarette smokers and in those conceiving after the use of ovulation induction drugs. An important predictor of preterm delivery is also the outcome of previous pregnancies; the risk is inversely related to gestation at previous spontaneous delivery, decreasing from 7% if the gestation was 16–24 weeks to 3% if 31–33 weeks, and to 0.6% if all deliveries were at term [14]. Screening in the first trimester, at 11–13 weeks by an algorithm combining maternal characteristics and obstetric history identified 38% of pregnancies resulting in spontaneous delivery before 34 weeks and 20% of those delivering at 34–36 weeks, at a false positive rate of 10% [15].

First trimester screening by a combination of maternal characteristics and obstetrical history, with the measurement of cervical length, can predict more than 50% of preterm births before 34 weeks’ gestation. This approach can provide patient-specific risks which can form the basis of the individualization of subsequent prenatal care and early intervention, either by performing a cervical cerclage or prophylaxis by intravaginal progesterone [15]. The majority of mortality and morbidity relates to early delivery before 34 weeks.

In women with short cervix at 20–24 weeks’ gestation, administration of progesterone reduced the risk of spontaneous early preterm delivery by about 45%. It has been demonstrated that progesterone is not as effective in women with a cervical length <10 mm as in those with a length of 10–20 mm [16].

## 6. Screening for Stillbirth

Stillbirth constitutes a major tragedy in obstetrics and unfortunately, with current prenatal care many cases are unpredictable and, therefore, not preventable. However, extensive evidence suggests that first, about 60% of antepartum stillbirths are observed in association with placental dysfunction [17], and second, more than half of such stillbirths can be effectively identified in the first trimester based on screening by a combination of maternal characteristics, maternal serum PAPP-A, the uterine artery pulsatility index (UtA-PI), and the ductus venosus pulsatility index [18,19].

The performance of screening is better for stillbirths secondary to impaired placentation compared to those that are unexplained, and in the impaired placentation group, the detection rate is higher for stillbirths that occur preterm than at term [19]. Values ≤ 0.42 MoM for PAPP-A, which corresponds to the 5th centile, increase the odds of having a stillbirth by a factor of 1.94 [20]. It has also been demonstrated that the risk of stillbirth increases 2.9-fold for every unit increase in the ductus venosus pulsatility index and 18-fold for every unit increase in the UtA-PI [18]. Pharmacological interventions in the high-risk group, by low-dose Aspirin starting at <16 weeks’ gestation could potentially improve placentation and reduce associated stillbirths by more than 50% [18].

## 7. Screening for Gestational Diabetes Mellitus

Early effective screening for gestational diabetes mellitus (GDM) can be performed at 11–13 weeks’ gestation by maternal characteristics and history in a multivariate logistic model with a detection rate of 80% [21]. The screening and diagnosis of GDM is traditionally delayed until the late second or early third trimester of pregnancy, because the diabetogenic effects of pregnancy increase with gestation. Subsequently, early appropriate dietary advice and pharmacological interventions might reduce maternal and perinatal complications.

The predictors of GDM are a previous history of GDM, a family history of a first- or second-degree relative with diabetes mellitus, maternal age, weight, height and racial origin, the method of conception and the birth weight of the neonate in the last pregnancy. It has been shown that for women with a previous history of GDM, the probability of developing GDM in the current pregnancy was very high and the contribution of risk factors other than weight was negligible [21]. Further improvement in the performance of screening, consists of the association of maternal characteristics with potentially useful biomarkers such as maternal serum adiponectin, visfatin, tissue plasminogen activator and sex hormone-binding globulin [22,23,24].

## 8. Screening for Placenta Accreta Spectrum Disorders

Effective screening for placenta accreta spectrum (PAS) can be performed in the first trimester. The maternal risk factors for pregnancies with potentially morbidly adherent placenta (MAP) were considered first, a history of uterine surgery, including Cesarean section or myomectomy that involved the opening of the uterine cavity, and second, a low-lying placenta, defined as the edge reaching to within 2 cm from the internal cervical os in the case of anterior placenta and reaching or covering the internal cervical os in the case of posterior placenta.

The diagnosis of MAP in the first trimester is considered if three of the following features are observed: a non-visible caesarean section scar; bladder wall interruption; thin retroplacental myometrium; intra-placental lacunar spaces; retroplacental arterial-trophoblastic blood flow; and irregular placental vascularization on 3D power Doppler [25]. The risk of PAS is higher in those with a low-lying placenta and previous uterine surgery. The markers for PAS most frequently seen in the first trimester are intra-placental lacunar spaces and a non-visible caesarean section scar [25].

Early diagnosis of MAP is important for patients who choose to continue with the pregnancy, because they could be referred for the management of the pregnancy and delivery in a hospital with expertise in this condition. This is particularly important in cases that develop the symptoms of uterine rupture during the second trimester and before fetal viability, because these patients have a high risk of maternal mortality or severe morbidity [26].

A summary of the available methods described above and clinically used in screening for pregnancy complications at 11–13 weeks is presented in Table 2.

## 9. Conclusions

Further research will expand the number of conditions that can be predicted from early pregnancy and define markers of disease that will improve pregnancy risk assessment. The early identification of low-risk and high-risk groups will help to define the best protocol for specific disorders in pregnancy and strategies for the prevention of disorders or their adverse consequences.

## Figures and Tables

**Table 1:** First trimester fetal anatomy assessment—Routine views [2].

• transverse section of the head to demonstrate the skull, midline echo and the choroid plexuses
• a mid-sagittal view of the face to demonstrate the nasal bone, midbrain and brain stem
• transverse views to demonstrate the orbits, upper lip and palate
• sagittal section of the spine to demonstrate the spine and overlying skin
• transverse section of the thorax
• color Doppler to assess the 4-chamber view of the heart and outflow tracks
• blood flow across the tricuspid valve (transverse view), ductus venosus (mid-sagittal view)
• transverse and sagittal sections of the trunk and extremities to demonstrate the stomach, kidneys, bladder, abdominal insertion of the umbilical cord, all the long bones, hands and feet

**Table 2:** Summary of methods used for screening at 11–13 weeks for pregnancy complications.

Condition Screened at 11–13 Weeks	Markers Used: Maternal and Fetal	Detection Rate
Trisomy 21	Maternal age Maternal characteristics Maternal serum PAPP-A and beta-hCG Nuchal translucency Nasal boneDuctus venosus flow Tricuspid flow	96%
Structural abnormalities	Standardized protocol for fetal ultrasound assessment	100% for: acrania, alobar holoprosencephaly, exomphalos, gastroschisis, body-stalk anomaly
Preeclampsia	Maternal characteristics Maternal history Mean arterial pressure Serum PlGF Uterine artery pulsatility index	90% for preeclampsia at <32 weeks
Small for gestational age	Maternal characteristics Maternal history Serum PAPP-A	60% of cases of SGA <10th centile at <32 weeks
Preterm birth	Maternal characteristics Obstetric history Cervical length	50% of cases at <34 weeks
Stillbirth	Serum PAPP-A < 0.42 MoM (<5th centile)	1.94 increase in relative risk of stillbirth
Gestational diabetes	Previous history Family history Maternal age, weight, height, and racial origin Method of conception Birth weight of neonate in last pregnancy	As high as 80%
Placenta accreta spectrum	Uterine surgery Low lying placenta Non-visible caesarean section scar, Bladder wall interruption Thin retroplacental myometrium Intra-placental lacunar spaces Retroplacental arterial-trophoblastic blood flow Irregular placental vascularization on 3D power Doppler	

PAPP-A—pregnancy-associated plasmatic protein-A; beta-hCG—beta human chorionic gonadotrophin; PlGF—placental growth factor; MoM—multiple of median.
